# Atypical Rapid Onset of Olmesartan-Induced Enteropathy with Recurrence After Rechallenging

**DOI:** 10.3390/diseases13070223

**Published:** 2025-07-18

**Authors:** Lila Bekkai, Aymen Ibn Majah, Laurine Verset, Lucas Jacobs, Charline Danneel, Okyay Elkilic, Frédéric Collart, Joëlle Nortier, Maxime Taghavi

**Affiliations:** 1 Nephrology and Dialysis Department, Brugmann University Hospital, Université Libre de Bruxelles (ULB), 1020 Brussels, Belgium; lila.bekkai@ulb.be (L.B.); aymen.ibn.majah@ulb.be (A.I.M.); lucas.jacobs@chu-brugmann.be (L.J.); frédéric.collart@chu-brugmann.be (F.C.); joelle.nortier@chu-brugmann.be (J.N.); 2 Pathology Department, Hôpital Universitaire de Bruxelles (HUB), Institut Jules Bordet, Université Libre de Bruxelles (ULB), 1070 Brussels, Belgium; laurine.verset@hubruxelles.be; 3 Pharmacy Department, Brugmann University Hospital, Université Libre de Bruxelles (ULB), 1020 Brussels, Belgium; charline.danneel@chu-brugmann.be; 4 Gastroenterology Department, Brugmann University Hospital, Université Libre de Bruxelles (ULB), 1020 Brussels, Belgium; okyay.elkilic@chu-brugmann.be

**Keywords:** enteropathy, diarrhea, olmesartan, acute kidney injury

## Abstract

Background: Olmesartan-induced enteropathy is a rare complication of a widely used angiotensin II receptor blocker. Patients usually present with chronic diarrhea and weight loss. Histologically, villous atrophy and intraepithelial lymphocyte infiltrates within the duodenum confirm the diagnosis. The prognosis is usually good after withdrawal of the offending drug. Case presentation: Here, we report the case of a 76-year-old woman who developed a severe form of Olmesartan-induced enteropathy complicated by acute kidney injury and acute recurrence after drug rechallenge. After definite cessation of the drug, the patient did not experience any gastrointestinal (GI) symptom recurrence after 6 months of follow-up. However, she experienced chronic kidney disease stage G3b. Histological analysis did not show any villous atrophy or intraepithelial lymphocyte infiltrates within the duodenum or the colon biopsy. Discussion and conclusion: This case highlights the broad spectrum of clinical manifestations and histological findings in Olmesartan-induced enteropathy. It also highlights the importance of rapid diagnosis in order to limit organ damage such as chronic kidney disease.

## 1. Introduction

Since the early 2000s, olmesartan has been a widely used antihypertensive and nephroprotective drug, which acts as an angiotensin II receptor blocker (ARB). Olmesartan-induced enteropathy (OIE) was first described in 2012 by Rubio-Tapia et al. as a “reversible sprue-like enteropathy” associated with the use of olmesartan [1]. Although the epidemiology is not well known, OIE is rare, with an estimated prevalence of 10 cases per 100,000 inhabitants [2]. Years after olmesartan initiation, patients usually complain of chronic diarrhea and weight loss, although nausea, vomiting, and gastroesophageal reflux disease have also been reported [3,4,5]. Typically, the duodenum histology of patients prone to these enteropathies shows the presence of villous atrophy, active inflammation, and intraepithelial lymphocyte infiltrate [6]. Some patients share the same characteristics except that their villous patterns are preserved (4). The prognosis is usually good after the withdrawal of the drug, and most patients are treated on an outpatient basis. However, some patients require emergency intervention [4,7]. 

Here, we report an atypical rapid onset of OIE without duodenum and colon histological lesions that relapsed after rechallenging the drug. The purpose of this case report is to highlight the broad spectrum of clinical and histological presentations of OIE as well as the potential risk of organ damage.

## 2. Case Presentation

A 76-year-old woman with a history of arterial hypertension and atrial fibrillation was daily treated with bisoprolol 5 mg, apixaban 5 mg bid, digoxin 0.125 mg, and olmesartan 20 mg. This last drug had been started two weeks before admission because of uncontrolled hypertension. The patient had no other medical condition. The patient was admitted to the emergency room with a deteriorated general status, acute onset of watery diarrhea along with multiple episodes of vomiting and hypotension, and 4 kg weight loss. She denied having fever, abdominal pain, recent travel, or dietary changes. Upon admission, her blood pressure was 80/60 mmHg. Laboratory tests performed at presentation detected mild inflammation and acute kidney injury (AKI) with mild hypokalemia, attributed to dehydration and diarrhea (Table 1). 

Renal ultrasound ruled out any obstructive cause of AKI. Stool cultures and parasitology were performed, which did not identify any enteropathogens. An etiological workup failed to find any infectious disease.

**Figure 1:**
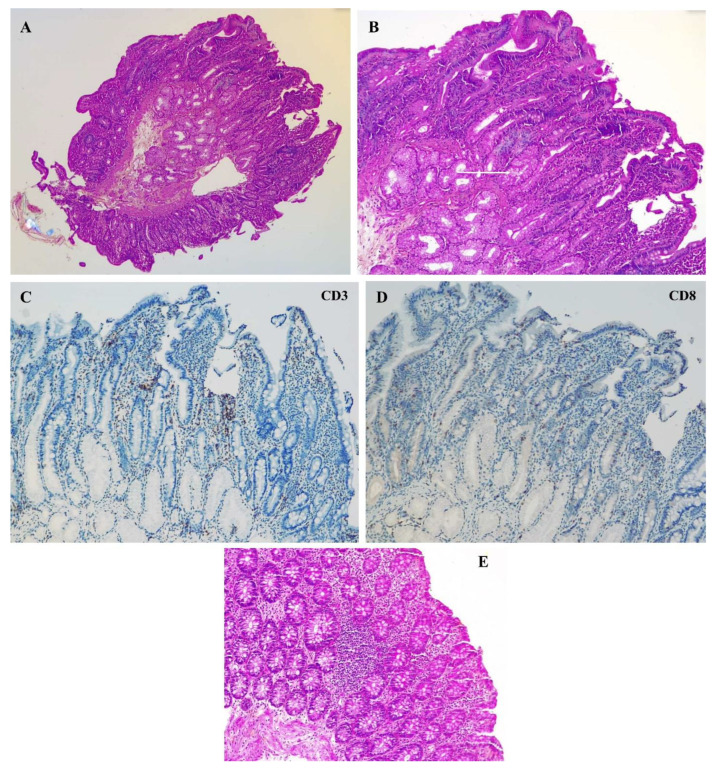
Histological findings within the duodenum and the colon. Panel (**A**): Duodenal biopsy fragment (hematoxylin and eosin stain, 5× magnification), showing non-atrophic duodenal mucosa, without evidence of intraepithelial lymphocytosis or significant increase in the inflammatory infiltrate. Panel (**B**): Duodenal biopsy fragment (hematoxylin and eosin stain, 10× magnification), showing non-atrophic duodenal mucosa, without evidence of intraepithelial lymphocytosis or significant increase in the inflammatory infiltrate. White arrow shows the presence of Brunner’s glands outside the submucosa. Panel (**C**,**D**): duodenal biopsy fragment (hematoxylin and eosin stain, 10× magnification), showing non-atrophic duodenal mucosa, without evidence of intraepithelial lymphocytosis or significant increase in the inflammatory infiltrate. White arrow shows the presence of Brunner’s glands outside the submucosa. Panel (**E**): Colon biopsy (hematoxylin and eosin stain, 10× magnification) showing no significant increase in the inflammatory infiltrate colonic mucosa. A focal lymphoid aggregate is observed, which may be seen under physiological conditions depending on the biopsy site. There is no increase in intraepithelial lymphocytes and no thickening of the basement membrane.

Histological examination was unremarkable without significant lymphocytic inflammatory infiltrate in the duodenum or villous atrophy. In the antrum and fundus, the glandular architecture was preserved with normal differentiation, but there was notable congestive edema in the antrum’s chorion without specific inflammation or atrophy. The ileum showed no significant abnormalities, with preserved villous architecture and no inflammatory changes. In the colon, findings included conserved crypt architecture with a mild, nonspecific lymphocytic infiltrate (Figure 1).

**Figure 2:**
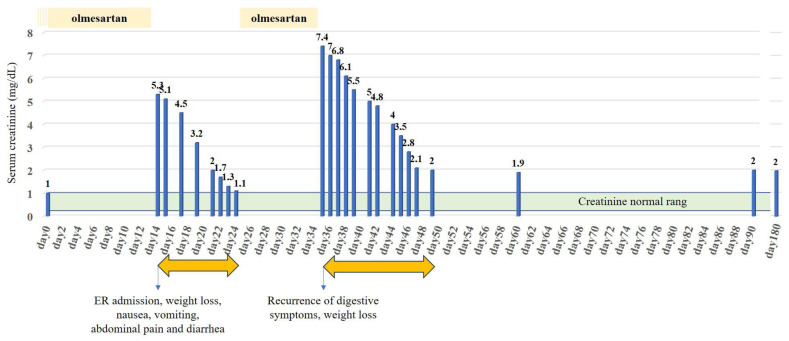
Time course of serum creatinine levels (blue bars) in relation to exposure to olmesartan. The green area represents the normal serum creatinine range. Orange arrows represent the two hospitalization periods in the nephrology department.

Olmesartan was withdrawn at admission, and GI symptoms as well as kidney function parameters improved rapidly. The patient was discharged 10 days after the admission, and the antihypertensive treatment was reintroduced (Figure 2).

Ten days after the patient was discharged, and before the planned follow-up consultation, she experienced a recurrence of GI symptoms and an even more severe AKI (Figure 2). Celiac serology was negative (transglutaminase IgA antibody and deamidated gliadin IgA and IgG antibodies). Again, the withdrawal of olmesartan led to the resolution of symptoms. Subsequently, OIE was diagnosed, and olmesartan was withdrawn with a complete resolution of diarrhea and the inflammatory state, and the patient was discharged from the hospital on day 10. No recurrence was observed during the 6-month follow-up. However, the patient did not fully recover her kidney function parameters and developed chronic kidney disease, stage G3b according to KDIGO (creatinine level 2.0 mg/dL, estimated glomerular filtration rate (eGFR) 35 mL/min/1.73 m^2^ according to the CKD-EPI equation).

## 3. Discussion

Enteropathy is a rare but significant complication of olmesartan. Other ARBs have been associated with OIE, such as telmisartan, irbesartan, valsartan, losartan, and eprosartan [2,8]. In 2019, Kamal A, et al. performed a systematic review by analyzing 248 cases of ARB-associated enteropathy published between 2012 and 2018. Among 233 cases reviewed, olmesartan was the drug mostly involved, with the remaining 15 cases suspected to be secondary to other ARBs [2]. Interestingly, after careful review of our patient’s medical file, the patient was treated with valsartan years before, without experiencing symptoms of enteropathy. 

OIE is characterized by GI symptoms that might be severe, resulting in severe dehydration and even acute kidney injury (AKI) in severe cases. Several case reports and case series reported AKI in patients with OIE [9,10]. Roca-Argente L, et al. reported 19 cases of OIE without previous CKD (mean eGFR before admission was 86.0 ± 17.4 mL/min/1.73 m^2^), of which 14 had AKI. AKI was classified according to the Acute Kidney Injury Network (AKIN) classification [11]. Eleven out of fourteen AKI episodes were classified as AKIN 2 (n = 2) and AKIN 3 (n = 9). Kidney biopsy was not performed, and AKI was attributed to dehydration. All the patients recovered from AKI, and no CKD complication was reported [12]. Also, the authors reported that a significant proportion of patients presented with hypokalemia was more prevalent in AKIN 3. Our patient similarly presented with severe AKI classified as AKIN 3, with concomitant hypokalemia. AKI was attributed to significant dehydration secondary to diarrhea and angiotensin II blockade, as urea fractional excretion and sodium fractional excretion were low. Recovery of kidney function was rapid once olmesartan was discontinued, which is consistent with findings across multiple studies showing that symptom resolution promptly occurs following drug cessation [2,3,4,5,9,12,13,14,15]. However, our patient did not fully recover from AKI after olmesartan was rechallenged, and ultimately, she developed CKD stage G3b at follow-up. 

According to the literature, OIE generally manifests after prolonged use of olmesartan, with a median of two years following the introduction of the drug [2]. Our patient presented with a rapid onset of symptoms within two weeks of olmesartan initiation. This accelerated presentation suggests a hypersensitivity reaction, possibly linked to individual predisposition. Our patient also experienced a recurrence of the condition when the ARB was reintroduced, leading to a more severe manifestation. This is notable because the relapse occurred in a shorter time frame, suggesting heightened sensitivity to ARBs after the initial episode. 

**Figure 3:**
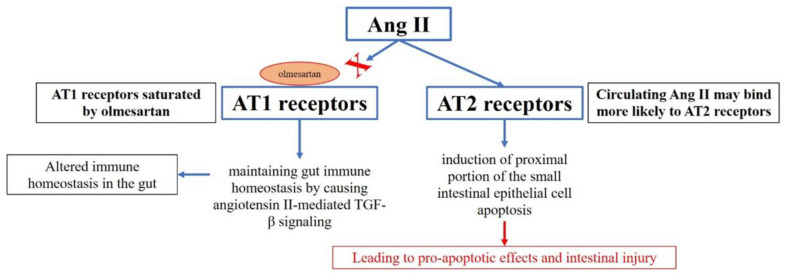
Hypothetical pathophysiology of olmesartan-induced enteropathy.

The pathophysiology of OIE is not fully elucidated, but a cell-mediated hypersensitivity reaction has been proposed. Olmesartan binds with more affinity to angiotensin II receptor type 1 (AT1 receptor), and AT1 receptors are saturated by olmesartan. Therefore, circulating angiotensin II may bind more likely to AT2 receptors. Interestingly, AT2 receptors are located in the proximal areas of the small intestine and are involved in the induction of intestinal epithelial cell apoptosis. It is hypothesized that stimulation of AT2 receptors in the setting of AT1 receptor blockade could lead to pro-apoptotic effects and intestinal injury, ultimately leading to OIE clinical manifestations [16,17]. Also, predisposition factors have been described, such as the association with the haplotype DQ2/DQ8 [18]. Figure 3 summarizes the pathophysiological hypothesis of OIE. 

Histological variability in OIE makes diagnosis challenging, with some patients demonstrating only subtle inflammatory changes or no obvious histological abnormalities, and some data suggest that certain patients exhibit unremarkable intestinal biopsies, complicating diagnosis [19]. In our patient, duodenum biopsies were largely unremarkable, aligning with findings that OIE can occur without the classic villous atrophy typically seen in celiac disease. Involvement of the colon has also been reported in OIE, which is not typically a primary site of pathology in OIE. Colon involvement has been actually documented in case series, supporting the idea that OIE may present with more widespread GI involvement than previously thought [20]. Intraepithelial lymphocytosis in ileum and colon biopsies is described in about 30% of OIE, as in our patient [4,21,22,23]. In our patient, colon biopsies revealed a focal lymphoid aggregate, without an increase in intraepithelial lymphocytes, which may be seen under physiological conditions depending on the biopsy site. The diagnostic workup requires multiple biopsies to be performed in different GI sites in order to capture the patchy distribution of OIE. Identification of the colon biopsy location is also of importance because it might influence the interpretation. This approach is well supported in the literature as necessary for an accurate diagnosis [4,18]. 

Early recognition of OIE is essential, yet it is often missed or delayed due to its nonspecific symptoms, such as diarrhea and weight loss [5], which overlap with other GI conditions. The episode of AKI in this patient underlines the need to consider OIE in patients presenting with unexplained renal impairment associated with GI symptoms. Symptomatic and histological improvement is described in most patients after discontinuation of the drug, highlighting the importance of early drug withdrawal to prevent further complications [1,19]. Table 2 summarizes the diagnostic traps leading to a delayed diagnosis of OIE in clinical practice.

Therapeutic management in OIE aims to stop the offending ARB, as seen in our patient. The recurrence of symptoms upon reintroduction of a similar ARB is an important consideration in clinical practice. Once a patient has developed OIE, rechallenge with any ARB should be avoided to prevent further adverse outcomes. The literature consistently warns of more severe and rapid recurrences upon re-exposure to ARBs, emphasizing the need for long-term follow-up and careful medication management [2]. This case aligns with these findings, as the patient’s second episode was not only faster in onset but also more severe, with greater renal impairment and dehydration, leading to CKD.

## 4. Conclusions

In conclusion, this case highlights the importance of maintaining a high index of suspicion for OIE in any patient on ARBs who presents with unexplained gastrointestinal symptoms, even in the absence of classic sprue-like lesions in the duodenum. The rapid onset and recurrence of symptoms in our patient as well as the intraepithelial lymphocytosis in colon biopsies highlight the variability in clinical presentation and the potential risk for further severe complications if the condition is not recognized early. Early recognition, drug withdrawal, and avoidance of re-exposure are crucial to prevent further adverse outcomes. Ongoing pharmacovigilance and clinician awareness are critical to ensure patient safety and minimize the risk of future episodes.

## Figures and Tables

**Table 1:** Laboratory findings upon admissions.

	1st Admission Values	2nd Admission Values	Normal Values
BLOOD			
Hemoglobin (g/dL)	14.3	15.6	13–18
MCV (fL)	90	93	80–100
Thrombocytes ×10^3^/µL	387	553	150–440
Leucocytes ×10^3^/µL	10.68	15.56	3.5–11
CRP (mg/dL)	32	24	<5
Urea (mg/dL)	437	127	17–48
Creatinine (mg/dL)	5.30	7.44	0.7–1.2
K/ HCO3 (mmol/L)	3.3/12	3.4/8	3.5–4.5/23–29
URINE			
Erythrocytes /µL	34	<12	<12
Leucocytes /µL	359	50	<10
Pathological cylinders	Absence	Presence (>6/2.25 µL)	Absence
Fractional excretion of urea (%)	11.9	13.2	
Fractional excretion of Sodium (%)	0.2	0.1	
Potassium (mmol/L)	9	40	<20

CRP, c-reactive protein; MCV, mean corpuscular volume.

**Table 2:** Diagnosis challenges leading to underdiagnosis of OIE in clinical practice.

- Rarity and underlooked disease [2]
- Heterogeneous clinical presentation, with a variety of symptoms ranging from mild enteropathy to severe diarrhea and malabsorption [4,5]
- Heterogeneous histological presentation, ranging from unremarkable intestinal biopsy to classical “sprue like enteropathy” with villous atrophy and intraepithelial lymphocyte infiltrate [6]
- Patchy distribution of histological lesions, requiring multiple biopsies from various gastrointestinal [4,18,20]

## Data Availability

Data are contained within the article.

## Abbreviations

The following abbreviations are used in this manuscript:

AKI	Acute kidney injury
ARB	Angiotensin II receptors blocker
AT	Angiotensin II receptor type 1
CKD	Chronic kidney disease
GI	Gastrointestinal
OIE	Angiotensin II receptor type 1 (AT1 receptor)
